# Does intranasal fentanyl provide efficient analgesia for renal colic in adults?

**DOI:** 10.11604/pamj.2015.20.407.6010

**Published:** 2015-04-24

**Authors:** Ahmed Belkouch, Saad Zidouh, Mostafa Rafai, Naoufal Chouaib, Rachid Sirbou, Anass Elbouti, Hicham Bakkali, Lahcen Belyamani

**Affiliations:** 1 Emergency Department, Mohamed V Military Hospital of Instruction, Faculty of Medicine and Pharmacy, Rabat, Morocco

**Keywords:** Emergency department, renal colic, intranasal fentanyl

## Abstract

**Introduction:**

Intranasal fentanyl provides rapid and powerful analgesia which is particularly interesting in patients without intravenous access. We propose to use it for analgesia in adults presenting renal colics.

**Methods:**

A prospective study was conducted from the 2nd January to February 2013 in our emergency department. Patients aged up to 18 years old who presented with renal colic were included in this audit. Patients were excluded if they had loss of consciousness, cognitive impairment, acute or chronic nasal problems. A formal written consent was obtained from patients. The research team was alerted by medical and nursing staff. A member of the research team would check with medical or nursing staff whether administration of Intra nasal (IN) fentanyl was required. It was administered at a pre-calculated dose of 1.5 mg/kg and 50 mg/ml concentration was used. Data was prospectively collected by one of the researchers at various intervals during the patient's presentation and recorded on a pre-formatted data sheet. Pain scores were collected at 5, 15, 30, 45 and 60 minutes following IN fentanyl using a visual analogue scale pain. Observations routinely collected for patients receiving IV opiates and any adverse events were also recorded.

**Results:**

23 eligible patientswere enrolled; median age was 51,3years. 47,8% were women and the mean weight was 73 kg. Median dose of IN fentanyl was 106 μg. Two patients have required morphinic analgesia despite having received adapted dose of IN fentanyl. The initial pain scores before IN fentanyl were high with a median of 82,2 mm (59-100). Five minutes after IN fentanyl administration the median pain score dropped to 48mm(36-63) and achieved the lowest score of 8mm(0-22) at 30 min. Pain scores were significantly lower at 5 min (P < 0.001) and at all subsequent time points (P < 0.001). No side effects were recorded.

**Conclusion:**

Intranasal fentanyl seems to be efficient for analgesia in adult patients with renal colic.

## Introduction

Renal colic is an intensely painful condition requiring rapid analgesic treatment. Opioids are commonly used intravenously to provide rapid pain relief. The objective of this study was to determine the analgesic efficacy and safety of intranasal single dose of fentanyl for patients presenting to the emergency department with renal colic.

## Methods

A prospective study of patients presenting with renal colic was conducted in the emergency department of the military hospital of instruction Mohammed V Rabat where an annual census of 30000 attendances are recorded. Enrollments were made from 2nd January 2013 to February 2013. Adults aged more than 18 years with acute flank pain due to renal colic were included in the study. Exclusion criteria were known allergy, contraindication to morphine, or any opioid analgesic; hemodynamic instability; fever (temperature ≥38°C (100.4°F' > 100.4°F)); evidence of peritoneal inflammation; documented or suspected pregnancy; known or suspected aortic dissection or aneurysm; use of any analgesic treatment within 6 hours before presentation to the Emergency Department; or previous study enrollment. Patients with known renal, pulmonary, cardiac, or hepatic failure, as well as those with renal transplantation, were also excluded. Patients were enrolled 24 hours a day and 7 days a week during the study period. Study eligibility was confirmed by a senior emergency medicine resident. Verbal consent was obtained from patients. Written consent was not obtained to minimize “time to analgesia” in the setting of acute extreme pain. Patients were given intranasal fentanyl at concentration of 50μg/ml and a dosage of 1,5μg/kg. Advantages of this concentration solution are standard availability in all Emergency Departments and lower cost. The intranasal solution was delivered via an atomizer (MAD100 Wolfe Tory Medical Inc.). Assessment of pain, using a 10 mm intervals visual analog scale pain (VAS), was performed at 5, 15, 30, 45 and 60 minutes following IN fentanyl. If the visual analogue scale was still over 4 after the first 5 minutes, we used intravenous morphine to procure quick relief. Data was prospectively collected by one of the researchers and recorded on a pre-formatted data sheet. During the study period, routine observations were noted in accordance with our institution's nursing protocols for the administration of narcotics. Parameters included were blood pressure, pulse rate, respiratory rate, and oxygen saturation every 5 minutes. Adverse events were also recorded. Comparison of patient assessed pain scores collected at 5, 10, 15 and 30 min following IN fentanyl administration were completed using the Wilcoxon signed-rank test. P values are 2 sided and were considered statistically significant if less than 0.05. All analyses were performed using the SPSS program (version 13.0; SPSS Inc., Chicago, IL, USA).

## Results

**Figure 1:**
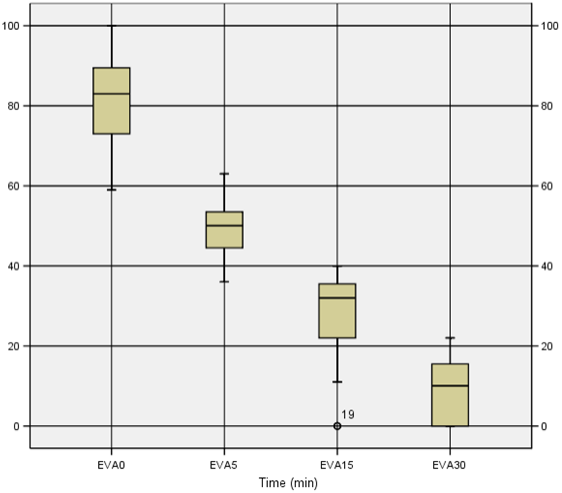
Pain scores at different moments

23 eligible patients were enrolled; median age was 51.3 years. 47.8% were women and the mean weight was 73 kg. Median dose of IN fentanyl was 106 μg. Two patients required morphinic analgesia despite having received adapted dose of IN fentanyl. The initial pain scores before IN fentanyl was given were high with a median 82,2mm (59-100) (Table 1). Five minutes after IN fentanyl administration the median pain score dropped to 48mm(36-63) and achieved the lowest score of 8mm(0-22) at 30min. Pain scores were significantly lower at 5 min (P <0.001) and at all subsequent time points (P < 0.001) (Figure 1). Oxygen saturation at baseline ranged from 97 to 99%, and only one patient had 94% 10 minutes after administration of IN fentanyl. For all our patients the blood pressure levels remained stable ranging between 123 and 143 mmHg for systolic BP and between 74 and 86 for diastolic BP. The mean heart rate at baseline was 95 (85-120), after pain relief, the mean heart rate at 15 min was 83 (60-100), and at 30 min it was 70 (56-90). Mean respiratory rate at baseline was 18 (16-24), at 15 min it was 15 (12-18) and remained stable.

**Table 1:** Patients and disease characteristics at baseline

Baseline variable	Value
Percentage of women(%)	47.8
Age (years)	51.3 (25-75) ± 13.4
Weight (kg)	73.2 (50-100) ± 14
Fentanyl dose administrated (μg)	106.5 (75-150) ± 21.8
Fentanyl dose per kg (μg/kg)	1.46 (1.16 -1.67) ± 0.14
Visual analogic assessment (mm)	82.2 (59-100) ± 11.5

## Discussion

Intranasal opioid administration has been studied widely in postoperative patients [1, 2] and burn patients [3, 4]. It has been also compared to intravenous morphine in pediatrics patients. In fact, an IV narcotic would be likely to be superior to an intranasal narcotic at 5 minutes because of the slight delay in absorption of intranasal fentanyl in comparison to IV administration of morphine. Intranasal fentanyl has been shown to have therapeutic serum levels in 2 minutes, reflecting the good venous outflow of nasal mucosa and bypassing the liver, avoiding hepatic first passage metabolism [5]. In the clinical setting, intranasal fentanyl can be administered promptly into the nasal cavity without the delays inherent in placing an IV. This mean of fentanyl administration could be part of the triage assessment by nursing staff in an effort to reduce “time to analgesia”. There were no significant adverse drug effects in our study. These findings were similar to that of other studies, one of them, a randomized, double blinded, placebo-controlled study showed that Intranasal fentanyl provided analgesia equivalent to that of IV morphine for children aged 7 to 15 years with acute fractures in the ED setting [1, 6–8]. INF has been shown to be equivalent or superior to morphine that is administered orally, intravenously, and intramuscularly and equivalent to intravenously administered fentanyl in providing pain control in children for many painful procedures [9]. We found the same results in our study. INF has a strong safety profile, it is easily available in the hospital setting, and does not require additional pharmacy compounding in its commercially available form. This strong evidence, in addition to the significant ease of administration and no requirement for intravenous line placement, make it a potentially superior option and/or adjunct treatment for acute and procedural pain control in children. We started using it within the framework of ongoing study in the patient's triage. When compared with current practices, it has the potential to improve patient and family satisfaction and could have further implications related to improved cost-effectiveness and quality of care [10]. The IN route offers more flexibility to the nursing staff; they can administer repeated doses to titrate against pain. In addition, it has the potential to be used in a patient controlled self-administered manner [5].

## Conclusion

The intranasal fentanyl can be a good option for analgesia in case of renal colic because it provides a quick pain relief and its use is safe. Confirmation of its benefits requires studies on larger cohorts.
